# Environmental factors driving the abundance of *Philaenus spumarius* in mesomediterranean habitats of Corsica (France)

**DOI:** 10.1038/s41598-023-28601-4

**Published:** 2023-02-02

**Authors:** Marguerite Chartois, Xavier Mesmin, Ileana Quiquerez, Sabrina Borgomano, Pauline Farigoule, Éric Pierre, Jean-Marc Thuillier, Jean-Claude Streito, François Casabianca, Laetitia Hugot, Jean-Pierre Rossi, Jean-Yves Rasplus, Astrid Cruaud

**Affiliations:** 1 CBGP, INRAE, CIRAD, IRD, Institut Agro, Univ Montpellier, Montpellier, France; 2 AGAP, INRAE, CIRAD, Institut Agro, Univ Montpellier, San Giuliano, France; 3 Conservatoire Botanique National de Corse, Office de l’Environnement de la Corse, Corte, France; 4 AgroParisTech, Paris, France; 5 Centre INRAE de Corse, San Giuliano, France

**Keywords:** Ecology, Entomology

## Abstract

*Philaenus spumarius* (*Ps*) is considered the main insect vector of the bacterium *Xylella fastidiosa* (*Xf*) in Europe. As such, it is a key actor of the *Xf* pathosystem on which surveillance and management strategies could be implemented. Although research effort has increased in the past years, the ecological factors shaping *Ps* abundance and distribution across landscapes are still poorly known in most regions of Europe. We selected 64 plots of 500m^2^ in Corsican semi-natural habitats in which we sampled nymphs and adults of *Ps* during three years. While local or surrounding vegetation structure (low or high scrubland) had little effect on *Ps* abundance, we highlighted a positive relationship between *Ps* abundance and the density of *Cistus monspeliensis* in the plots. We also found larger populations of *Ps* in cooler and moister plots. The pattern of host association highlighted here is unique, which calls for more studies on the ecology of *Ps* in Europe, to help designing surveillance and management strategy for *Xf*.

## Introduction

The meadow spittlebug *Philaenus spumarius* (Linnaeus, 1758) (Hemiptera: Aphrophoridae) (*Ps*) is considered the main vector of the plant pathogen *Xylella fastidiosa (Xf)* in Europe^1,2^. Research effort on its biology and ecology has recently increased, generally with the aim of proposing management strategies. However, effort is unequal among regions and habitats and it is still unknown whether environmental drivers of abundance and distribution of *Ps* (e.g., climate and host plants) are similar between different ecosystems and/or geographical regions.

Humidity and temperature are important drivers of the development and the geographic distribution of *Ps*^2,3^. Overall, studies have either addressed physiological or phenological responses of *Ps* to temperature and humidity at local spatial scales, or climate preferences at larger scales^4^, notably using species distribution models^5–7^. Regional studies investigating the effects of climatic conditions on populations of the meadow spittlebug are still scarce, with the exception of works by Drosopoulos and Asche^8^ in Greece, Godefroid and Durán^9^ in southern Spain, and Karban and Strauss^10^ in California. Authors agree that humidity and temperature affect the development time of immature stages of *Ps*^3,11,12^. While exact values vary, cool and moist conditions appear more favorable, with minimum temperatures for egg hatching and larval development ranging from 4 to 10 °C^3,11,13^. No data are available regarding maximum temperatures but nymphal development is still observed at 27 °C^3,11^. Humidity promotes hatching and survival of nymphs that need sufficient sap flow to feed. Nymphs produce a foam that covers them and to which they owe the name "spittlebugs"^3^. This foam likely protects them from predators and dehydration^14^. In this regard, extreme or prolonged summer droughts drastically reduce *Ps* populations^10,15^. Moreover, shift in host plants from dry vegetation to less water-stressed shrubs and trees or migration toward coolest places (e.g., near rivers) have been documented, showing that humidity and temperature play a major role in the ecology of this insect^3,15–19^.

Trophic interactions between insect vectors and their host plants can shape the epidemiology of plant diseases caused by *Xf*. In this regard, with more than 1000 host plant species in 75 families^3,20,21^, the highly polyphagous^2,3,22^ *Ps* is a key actor of the *Xf*-pathosystem. Several studies have explored local host associations of *Ps* in olive groves in Italy^23,24^, Portugal^25^, Spain^19^ and Greece^26^, where nymphs mostly feed on Asteraceae, Fabaceae and Apiaceae. However, no study has explored the link between the local abundance of host plants and the degree of polyphagy of *Ps*. Summer migration of adults from ground cover to olive foliage, linked with drying of herbaceous plants has been observed in southern Italy^2,21^. In particular, adults of *Ps* were observed in the vicinity of olive groves, from May to August, on *Quercus ilex*, *Quercus x crenata*, *Pistacia lentiscus*, *P. terebinthus, Hedera helix* and *Myrtus communis*^17,21,24^. In the Apulia region, *Ps* adults have also been found on *Pinus halepensis* from May to September and have been reported once on *Ulmus minor* in October^17^. On the other hand, in Belgium, Hasbroucq et al.^27^ reported adults of *Ps* on Ranunculaceae, *Prunus* sp., *Rubus* sp., *Crataegus* sp., *Alnus* sp., *Picea* sp., *Quercus* sp., *Salix* sp., *Carex* sp. and *Urtica* sp.*.* Recent studies performed in Corsica, France^5,28,29^ suggest that *Ps* has affinity with *Cistus monspeliensis*, at least locally. Provided this holds on a larger scale, *Ps* populations are expected to grow with *C. monspeliensis* cover^30^. However, it should be evaluated whether insect load (i.e., insect population size per plant unit) will increase, decrease, or be uncorrelated with host plant cover^30,31^. The degree of preference for a plant species over its neighbors seems to play a key role in this relationship and is often negative (i.e., insect load decreases with plant density) when the focal plant species is preferred over its neighbors^31^. This phenomenon is known as "resource dilution" and given that it is usually observed in specialist species^32^, it potentially does not contribute to determining the preference in a generalist insect such as *Ps*.

Another important aspect to consider within a vector-borne pathosystem is the dispersal ability of vectors. In flight mills, the average distance flown by *Ps* adults vary from one to five hundred meters^33,34^. In olive groves and meadow landscapes, the median distance covered during *Ps* adult life was assessed at 374 m and 507 m, respectively^35^. Although limited, this dispersal ability creates connection between habitats (e.g., grassland, meadow, forest, crops etc.) that are within a radius of some hundreds of meters. Consequently, *Ps* population size at a given site is likely to be influenced by the suitability of the surrounding habitat. Accordingly, population size of *Ps* has been shown to correlate positively with the surface of olive groves^36,37^ in a 125 m-radius buffer surrounding studied plots, while it negatively correlated with the surface of vineyards^36^. *Ps* density was also found to be lower in sites with high forest cover^38^. Considering landscape composition around study sites to investigate drivers of *Ps* population size seems therefore crucial.

In the present study, we aimed at comparing *Ps* abundance in plots distributed along a gradient of climate conditions and characterized by contrasted vegetation structure, and various landscape contexts in mesomediterranean areas of Corsica. We addressed five research questions stemming from our review of the literature: (1) Are populations of *Ps* larger in areas densely covered with *C. monspeliensis*?; (2) Does *Ps* load on *C. monspeliensis* plant increase, decrease or remain steady with *C. monspeliensis* cover?; (3) Are populations of *Ps* larger in cooler and moister plots, especially during the hot and dry seasons?; (4) Does vegetation structure (tree cover versus open areas) impact the abundance of *Ps*, especially during the hot season? and (5) Does vegetation structure of the surrounding environment impact the abundance of *Ps*?.

## Material and methods

### Sampling plots

**Figure 1:**
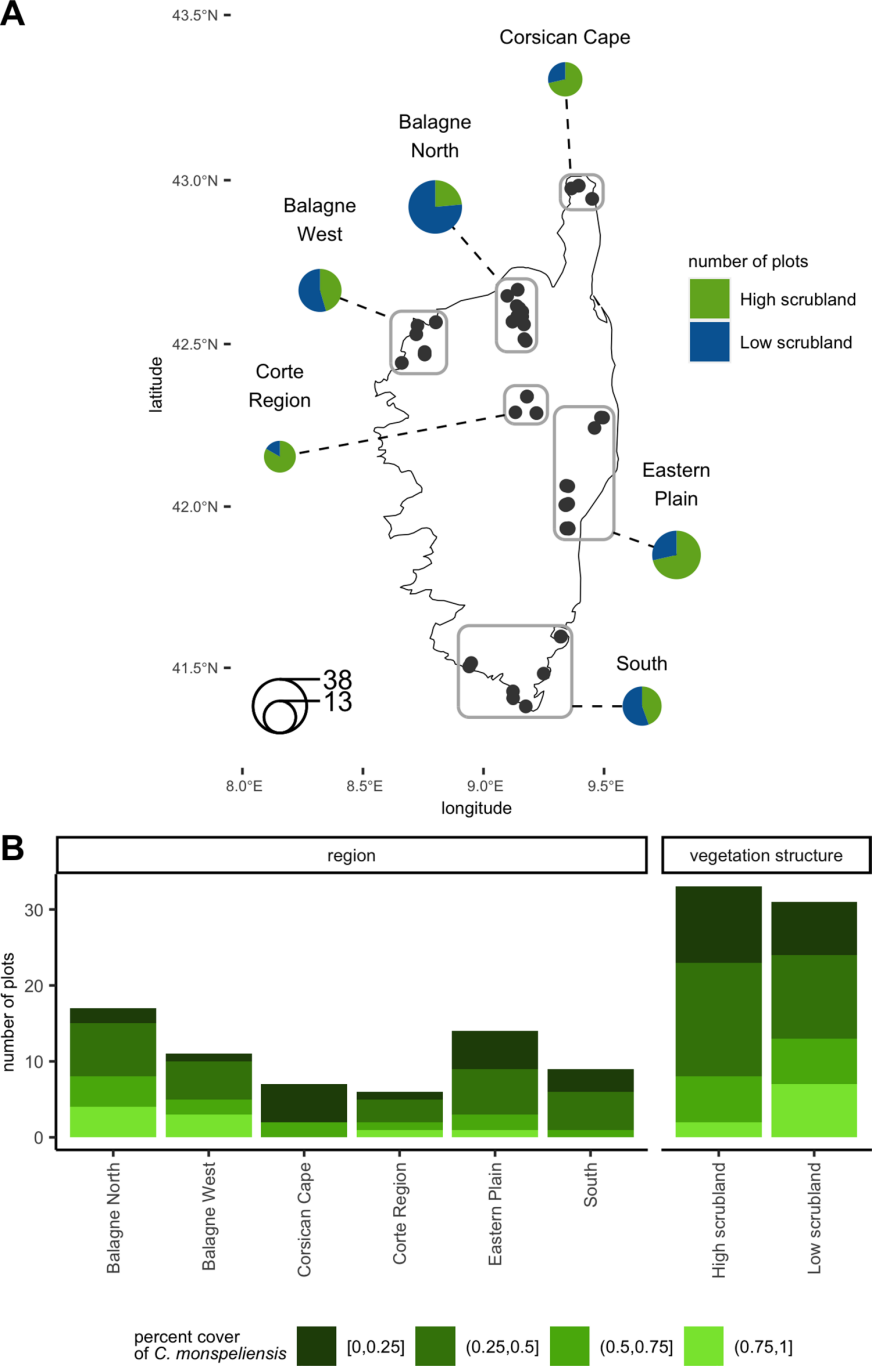
Sampling scheme. (**A**) Distribution of plots and vegetation structure. The size of the circles is proportional to the number of plots. (**B**) Percent cover of *C. monspeliensis* in each region and for each type of vegetation structure: high scrubland (tree cover exceeding 25%) and low scrubland (shrub and herbaceous formations with tree cover below 25%).

Sixty-four plots of 500 m^2^ were distributed in different regions of Corsica (Fig. 1; Supplementary map) along two gradients: one gradient of *C. monspeliensis* cover (from 0 to 95%) and one elevation gradient (from 6 to 595 m above sea level). These two uncorrelated gradients (Spearman’s rank correlation test p-value: 0.7586) allowed us to independently assess the effects of vegetation and climate on *Ps* abundance.

### Sampling period and frequency

First, a phenological survey of populations of *Ps* was performed bi-weekly on two plots (CAN-01 and CAN-02, Table S1) to identify periods during which densities of nymphs and adults were the highest. Based on this phenological survey, field work was conducted on all sampling plots in April (highest density of nymphs); June (highest density of young adults) and October (breeding season). Conducting a field survey for a fortnight at peak abundance indeed contributed to minimize differences in abundance between plots. These three sampling sessions were repeated during three consecutive years (2018, 2019 and 2020).

### Within-plot vegetation structure

All studied plots were located in the scrubland vegetation, a common vegetation type at the mesomediterranean level^39,40^ (see botanic inventory in Table S1 for details). The percent cover of live *C. monspeliensis* was visually estimated on each plot at each sampling session. The median values over all sessions were used in subsequent analyses for all but two plots (VENT-03 and VENT-04) on which vegetation was cut by municipal services to prevent bushfires. For these two plots, *C. monspeliensis* covers measured at each session were used instead.

Vegetation structure within plot was retrieved from the OCS GE database (© IGN–2022, https://geoservices.ign.fr/ocsge) provided by the French National Institute of Geographic and Forestry Information. This database includes land cover and land use, photo-interpreted from 50 cm-pixels orthophotographs, in the form of a polygon database whose contours are positioned with a precision of *ca.* 1 m. Vegetation structure within plot was coded as follows: "high scrubland" (when tree cover exceeded 25%); number of plots n = 33, and "low scrubland" (shrub and herbaceous formations with tree cover below 25%; n = 31; Fig. 1; Table S1; Supplementary map).

### Vegetation structure around plots

Vegetation structure around plots was also characterized through the OCS GE database. Based on *Ps* dispersal ability reported by Bodino et al.^35^ in natural conditions, we calculated the proportion of each type of vegetation structure within a radius buffer zone of 374 m, which was drawn around the plot but excluding the plot itself (see Supplementary map). It is noteworthy that results were unchanged with a radius buffer zone of 500 m (data not shown). Four vegetation structure were identified: "high scrubland", "low scrubland" (see previous section), "unvegetated" (artificialized areas, bare ground, water and sea surfaces), and "orchards" (tree or woody vines cover exceeding 25% with agricultural use, i.e., mainly olive groves, *Citrus* groves and vineyards).

Vegetation structure around plots was then analyzed by a Principal Component Analysis (PCA). The proportion of each of the four vegetation structures within each buffer was used as input for the PCA (Table S1). The PCA was performed using the R package ade4^41^. Scores of each plot on the first (PC1_landscape_) axis and second (PC2_landscape_) axis of the PCA were used as input variables in all generalized linear mixed models (GLMMs).

### Abundance of *P. spumarius*

In a preliminary experiment, we counted the number of nymphs in 20 randomly selected foams on each plot. An average of 1.24 nymphs per foam was found (± 0.57 standard deviation, Fig. S1) and this number did not differ significantly among plots (minimum p-value of Dunn test for multiple comparisons of means = 0.09, Fig. S1). Thus, we considered that the number of foams could be used as a good proxy for the number of nymphs and nymph's abundance was defined as the number of foams counted in 10 min in the vegetation of the plot (herbaceous, shrub, tree foliage of lowest branches, undergrowth) by the same operator.

Adult abundance was defined as the number of adults collected by two operators sweep netting the vegetation for 10 min, a time window sufficient to sample almost all the vegetation present in the plot (i.e., herbaceous, shrub, lowest branches of trees, and undergrowth vegetation). Sweep netting consisted in periods of 30 s of alternate backhand and forehand strokes with the net followed by 10–30 s of collection of adults with mouth aspirators. Sweeping the vegetation over a longer period would have increased risks of missing the target species in the net among plant debris and non-target arthropods. At any rate, after 10-min, adults collected in the mouth aspirators were counted and released afterward to preserve local insect populations.

To ascertain that foams/adults were correctly identified on sight, nymphs used to estimate the average number of nymphs per foam in the preliminary experiment and about 30 adults collected in the vicinity of each plot every June and October were killed using ethyl acetate, stored in 75° ethanol, and brought to the laboratory. We collected a total of 11,018 adults and 1911 nymphs. These specimens were identified to species level in the lab using a binocular microscope, the identification key of Biedermann and Niedringhaus^42^; illustrations from Stöckmann et al.^43^ and the Arthemis database (https://arthemisdb.supagro.inrae.fr^44^). All nymphs (1911) and 11,003 adults (99.9%) were confirmed as *Ps*. The remaining 0.1% of adults (N = 15), were re-identified as *Neophilaenus campestris.* Given this negligible error rate, counts based on identification on sight in the field were considered as reliable.

### Ecological drivers of *P. spumarius* abundance using bioclimatic data (GLMM1 models)

A first set of GLMMs (hereafter referred to as GLMM1 models) was used to assess the effects of percent cover of *C. monspeliensis*, climate, plot vegetation structure and surrounding vegetation structure on *Ps* abundance. Three GLMMs were built, one for each sampling month: GLMM1-April (nymphs), GLMM1-June (young adults) and GLMM1-October (breeding adults).

In this set of GLMMs, climate was described by a PCA performed on 19 bioclimatic variables (Table S1)^45^ retrieved from the SAFRAN model from Météo France^46^, which interpolates data measured several times a day by a network of over 1000 meteorological stations spread over the French territory. SAFRAN provides daily data of temperature (2 m above ground) and precipitation interpolated at a resolution of 8 km. Bioclimatic variables were derived from the monthly minimum, maximum, mean temperature and mean precipitation values, and were computed over the whole sampling period (2018–2020). They represented annual trends, seasonality, and extreme values of annual climate conditions. As no strong message emerged from the literature about the bioclimatic factors driving *Ps* abundance, we used all 19 bioclimatic variables. Scores of each sampling plot on the first axis (PC1_bioclim_) and second axis (PC2_bioclim_) of the PCA (Table S1) were used as input variables for all three GLMM1 models. The 19 bioclimatic variables were computed for all plots using the function biovars of the R package dismo^47^ to the three-year mean values of monthly temperatures (minimum and maximum) and precipitations (sum).

Structure of GLMM1 models, following glmmTMB notation was:

$$Ps\mathrm{\,\,abundance \,\,on\,\, month\,\, m }\sim \mathrm{ poly}\left(\mathrm{\%cover \,\,of \,\,Cm},\mathrm{ degree }3\right)+\mathrm{plot\,\, scores \,\,on\,\, PC}{1}_{\mathrm{bioclim}}+\mathrm{plot \,\,scores \,\,on\,\, PC}{2}_{\mathrm{bioclim}}+\mathrm{local \,\,vegetation\,\, structure}+\mathrm{plot\,\, scores \,\,on \,\,PC}{1}_{\mathrm{landscape}}+\mathrm{plot \,\,scores \,\,on\,\, PC}{2}_{\mathrm{landscape}}+\mathrm{year}+\left(1|\mathrm{id}\right)+ \left(1|\mathrm{observation}\right),\mathrm{ family}=\mathrm{nbinom}1(\mathrm{link}="\mathrm{log}")$$

where Cm, (1|id) and (1|observation) respectively stand for *C. monspeliensis*, random effect on the identifier of the plot and observation-level random effect, and $m\in \left\{April, June, October\right\}$.

### *P. spumarius* load per host plant unit* C. monspeliensis *cover (GLMM2 models)

A second set of models (hereafter referred to as GLMM2 models; one model per sampling month) was built with the same structure as GLMM1 models but the response analyzed was the *Ps* load per unit cover of *C. monspeliensis*. To do that we used rate models^48^, adding an offset—$offset(\mathrm{log}\left(\%cover \,\,of \,\,Cm\right))$—in the glmmTMB formula reported above. It should be noted that the response analyzed was only a proxy of *Ps* density on *C. monspeliensis*: we did not record the plant host of every *Ps* sampled. This proxy is thus slightly overestimating of the actual density, as some (but not much) individuals were collected on other host plants. Only significant variables of each GLMM1 models were included in corresponding GLMM2 models and only the 61 plots where *C. monspeliensis* was present were analyzed.

### Ecological drivers of *P. spumarius* abundance using seasonal climate data (GLMM1bis models)

A last set of GLMMs (hereafter referred to as GLMM1bis models) was used to check the robustness of the trends observed with GLMM1 models. They only differed from GLMM1 models in the way the effect of climate was tested. In GLMM1bis models (one for each sampling month), climate was described by a set of seasonal variables: average day temperatures (minimum, mean and maximum), sum and maximum day precipitations over the 1 or 2-month period preceding field surveys, all averaged over the 3 years of the study (10 climate descriptors, Table S1). A PCA was performed for each sampling month: April, June and October and scores of each plot on the first (PC1_seasonal_) and second (PC2_seasonal_) axis of the PCA were used for each corresponding GLMMs (GLMM1bis-April, GLMM1bis-June, GLMM1bis-October). This analysis is complementary to that of GLMM1 models because it relies on raw climate data (instead of elaborated bioclimatic variables) retrieved on a relevant period with respect to *Ps* phenology. The structure of GLMM1bis models was the same as for GLMM1 models. No significant differences between the results of GLMM1 and GLMM1bis models were found. Therefore, we did not use seasonal climate data to analyze *Ps* load per unit *C. monspeliensis* cover.

### Modelling framework

All GLMMs were built with the R package glmmTMB^49,50^ using linearly parameterized negative binomial distributions ("nbinom1"^51^). To account for repeated measures on the same plot, we added a random effect on the identifier of the plot^48^. An observation-level random effect was also added to correct overdispersion^52^. Year was included as an experimental design fixed effect because the number of factor levels was below 5^53^. As preliminary data analyses suggested a nonlinear relationship between *Ps* abundance and percent cover of *C. monspeliensis*, quadratic and cubic components of this variable were added to GLMMs. We used the DHARMa^54^ and performance^55^ packages to detect possible significant deviations from model assumptions (normality of residuals, homoscedasticity, the absence of collinearity between factors and the absence of highly influential data points by calculating Cook’s distance). To explore the results of the fitted GLMMs, type II analyses of deviance (R package car^56^) and post-hoc pairwise comparisons of factor levels were performed (R package emmeans^57^ and multcomp^58^).

## Results

For all correlations mentioned in the text, p-values are below 0.005. Details on χ^2^, df and exact p-values are available in Table S2.

### Spatiotemporal variability of the abundance of *P. spumarius*

**Figure 2:**
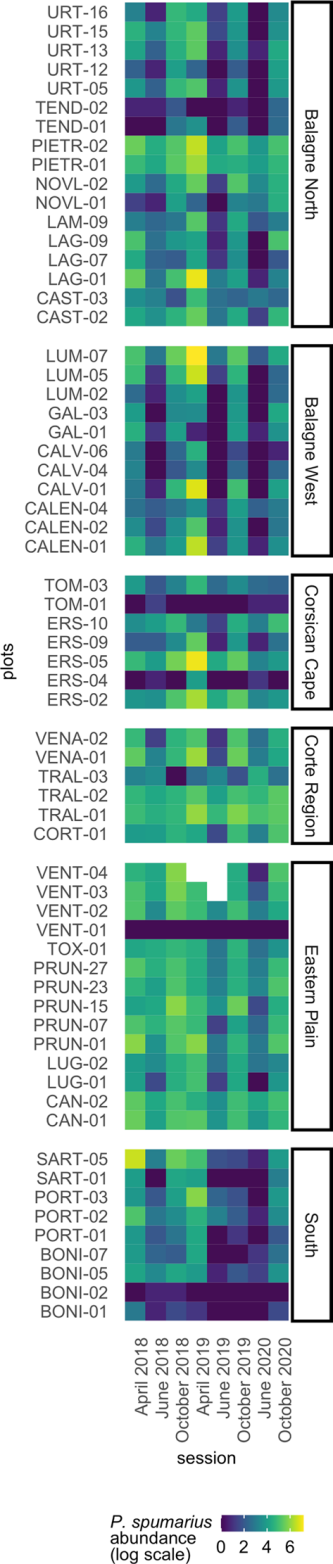
Abundance of *P.* *spumarius* and climate data. Spatiotemporal variability of *Ps* abundance (log scale). Rows show plots and columns show sampling sessions. White cells show NAs (vegetation cut).

A total of 19,808 foams (used as a proxy for nymphs, see methods) and 18,645 adults of *Ps* were counted during our three-year survey. Spatial and temporal variability of *Ps* abundance is illustrated in Fig. 2 (raw data are provided in Table S1). No data was collected in April 2020 because of restrictions due to the Covid-19 pandemic.

### Structure of vegetation surrounding sampling plots

**Figure 3:**
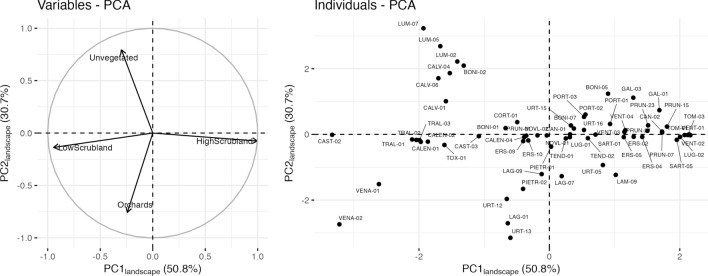
Principal component analysis of the proportion of each land cover type. (**A**) Correlation circle. (**B**) Projection of sampling plots in the first factorial plan.

PC1 and PC2 of the PCA performed on surrounding vegetation structure accounted for 50.8% and 30.7% of the variability, respectively (Fig. 3). PC1_landscape_ represented a gradient of vegetation density and opposed plots located in high scrubland landscapes (tree cover above 25%; correlation to PC1_landscape_: 0.997) to plots located in low scrubland landscapes (correlation to PC1_landscape_: −0.945). The area covered by high scrubland in the landscape was therefore strongly negatively correlated with the area covered by low scrubland. PC2_landscape_ opposed plots located in landscapes with large unvegetated areas (correlation to PC2_landscape_: 0.794) to plots located in the vicinity of orchards (correlation to PC2_landscape_: −0.756). Plots were first separated according to the scrubland structure (high or low) in their vicinity. Then, rarer elements of the landscape, unvegetated soils and orchards, were captured on PC2_landscape_ which distinguished plots with more rocky soil and distributed on the coast (e.g., LUM-07, CALV-04, BONI-02) from plots located in the Balagne region where the landscape is historically rich in olive orchards (e.g., LAG-01, URT-13).

### Ecological drivers of *P. spumarius* abundance using bioclimatic data (GLMM1 models)

**Figure 4:**
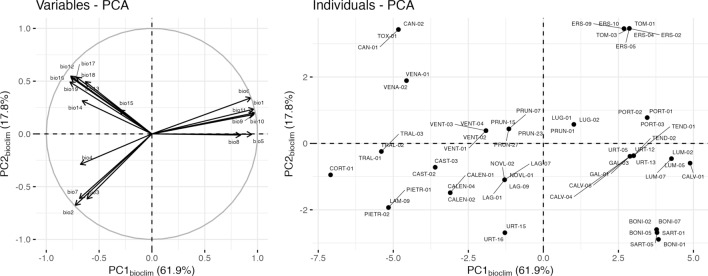
Principal component analysis of the 19 bioclimatic variables computed over the 2018–2020 period. (**A**) Correlation circle. Variable abbreviations are as follows: bio1 = annual mean temperature, bio2 = mean diurnal range, bio3 = isothermality (bio2/bio7) (× 100), bio4 = temperature seasonality, bio5 = maximum temperature of warmest month, bio6 = minimum temperature of coldest month, bio7 = temperature annual range (bio5-bio6), bio8 = mean temperature of wettest quarter, bio9 = mean temperature of driest quarter, bio10 = mean temperature of warmest quarter, bio11 = mean temperature of coldest quarter, bio12 = annual precipitation, bio13 = precipitation of wettest month, bio14 = precipitation of driest month, bio15 = precipitation seasonality (coefficient of variation), bio16 = precipitation of wettest quarter, bio17 = precipitation of driest quarter, bio18 = precipitation of warmest quarter, bio19 = precipitation of coldest quarter. (**B**) Projection of sampling plots on the first factorial plan. Raw data are provided in Table S1.

The transformation of the 19 bioclimatic variables through the PCA yielded two synthetic variables, PC1 and PC2, which supported 61.9% and 17.8% of the climate variability, respectively (Fig. 4). PC1_bioclim_ opposed plots with high temperatures and low precipitations to plots with high precipitations and low temperatures. The variables exhibiting the largest scores upon PC1_bioclim_ were the temperature of the warmest month and of the wettest quarter (right side of the axis, Fig. 4) and the temperature seasonality and the precipitation of the driest month (left side of the axis, Fig. 4). PC2_bioclim_ opposed plots with high seasonal climate variations to plots with low seasonal climate variations. The bioclim variables that displayed the largest scores on PC2_bioclim_ were the diurnal and annual temperature ranges (the "high seasonal variation" end of the gradient), and many equally projected precipitation-related variables in the positive direction (the "low seasonal variation" end of the gradient). Examples of raw climate values of the most contrasted plots according to the PCA are given in Table S3.

**Figure 5:**
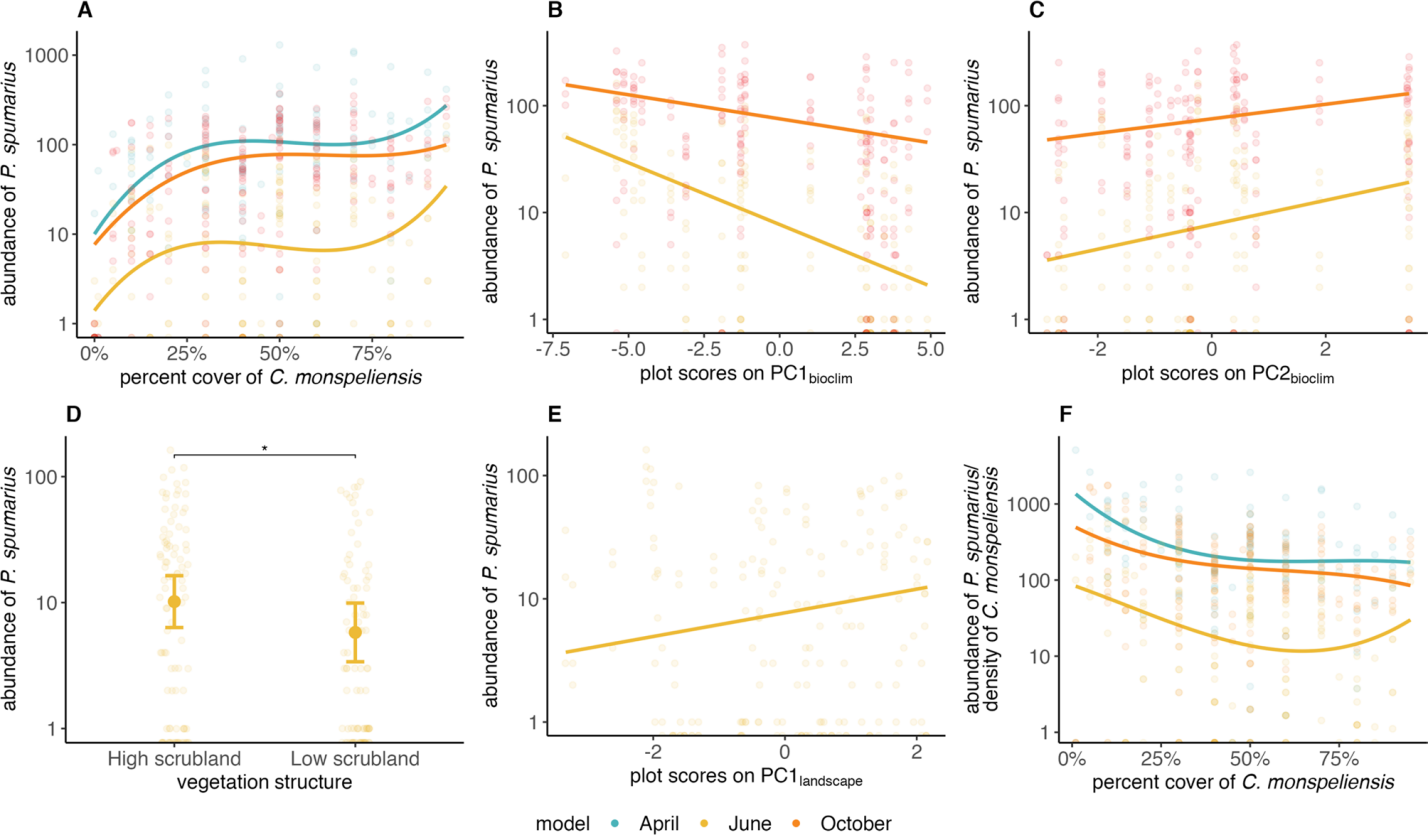
Scatterplots of raw data, regression lines and model prediction for pairwise associations between *P. spumarius* (*Ps*) abundance and fixed effects (GLMM1: panels (**A**) to (**E**), GLMM2: panel (**F**)). (**A**) *Ps* abundance and percent cover of *Cistus monspeliensis*. (**B**) *Ps* abundance and plot scores on PC1_bioclim_ (which rises with increasing temperature and decreasing precipitation). (**C**) *Ps* abundance and plot scores on PC2_bioclim_ (which rises with decreasing climate seasonality). (**D**) *Ps* abundance as a function of vegetation structure observed in the plot: high scrubland (tree cover exceeding 25%); low scrubland (shrub and herbaceous formations with tree cover below 25%). (**E**) *Ps* abundance and plot scores on PC1_landscape_ (which opposed landscapes with tree cover above 25% (high scrublands) at positive scores to landscapes with open areas and tree cover below 25% (low scrubland) at negative scores). (**F**) *Ps* abundance per host plant and percent cover of *C. monspeliensis*. Colors: sampling month. Error bars on scatterplots: 95% confidence intervals. Points: raw data; lines: regression curves from models. All correlations have p-values < 0.005. All details on significance tests and R2 of the regression lines are given in Table S2.

Models showed that *Ps* abundance was positively correlated with the percent cover of *C. monspeliensis* for each sampling month (Table S2). This correlation was characterized by a strong rise in *Ps* abundance for *C. monspeliensis* cover ranging from 0 to 25%, then abundance levelled off and finally rose again for *C. monspeliensis* cover exceeding 80% (Fig. 5A). Climate had a significant effect only on the abundance of adults (June and October)*.* The abundance of adults decreased with sample plot scores on PC1_bioclim_ (Fig. 5B). Therefore, *Ps* abundance increased with decreasing temperatures and increasing precipitations. Abundance of adults also increased with the scores of sample plots on PC2_bioclim_, that is with decreasing seasonal contrast in climatic conditions (Fig. 5C). Local (i.e., in plot) and surrounding vegetation structure correlated with *Ps* abundance only in June. Plots with high scrubland vegetation hosted higher abundances of young adults than plots with low scrubland vegetation (Fig. 5D), but vegetation structure of the plot had no effect on the abundance of nymphs or adults during the breeding season (Table S2). Abundance of young adults significantly increased with sample plot scores on PC1_landscape_ (Fig. 5E), which means that abundances of *Ps* was higher when tree cover in surrounding vegetation was higher. Contrastingly, structure of landscape vegetation had no effect on the abundance of nymphs or adults during the breeding season (Table S2). In all years, adult abundance was higher in October than in June (Figs. 4, S2).

### *P. spumarius* load per unit* C. monspeliensis* cover (GLMM2 models)

The effects of climate, local and landscape vegetation structures observed in GLMM1 models were identical to those observed with GLMM2 models (Table S2). In addition, for all sampling months, *Ps* load per unit *C. monspeliensis* cover was negatively correlated with *C. monspeliensis* percent cover (Fig. 5F).

### Ecological drivers of *P. spumarius* abundance using seasonal climate data (GLMM1bis models)

The PCA performed on the datasets associated to each date yielded two principal axes (PC1 and PC2) that accounted for 72.8% and 14.9% (April), 71.2% and 15.4% (June), and 62.3% and 31.8% (October) of the climate variability, respectively (Fig. S3). For the three PCA, PC1 opposed plots with high temperatures to plots with high precipitations and PC2 opposed plots with high temperatures and precipitations to plots with low temperatures and precipitations.

As in GLMM1 models, there was a positive correlation between *Ps* abundance and *C. monspeliensis* cover, with similar sigmoid pattern (Table S2; Fig. S4A). The abundance of adults increased with plot scores on PC1_seasonal_ in June and October (Fig. S4B), that is with increasing precipitations and decreasing temperatures. Only in June, abundance of *Ps* decreased with plot scores on PC2_seasonal_ (Fig. S4C), that is with decreasing precipitations and temperatures. Finally, neither local nor landscape vegetation structure had a significant effect on *Ps* abundance in GLMM1bis models.

## Discussion

As for our first question, we found that *Ps* population size increased (although not linearly) with *C. monspeliensis* cover in mesomediterranean semi-natural habitats of Corsica. In addition, this relationship holds at both nymphal and adult stages, demonstrating that the affinity of *Ps* for *C. monspeliensis* goes beyond previously described local trends^28,29^. To answer the second question, we did not use the number of *Ps* found on each *C. monspeliensis* plant but added instead an offset to the model, which was equivalent to using the number of *Ps* divided by the area covered by *C. monspeliensis* in each plot as the output variable. Using this proxy, we found that *Ps* load on *C. monspeliensis* decreased with *C. monspeliensis* cover, especially from 0 to 50% plot cover. This pattern is expected when an insect has preference for one plant species over all its neighbors^31,32^. Insect load per *C. monspeliensis* plant was maximal when *C. monspeliensis* plants were scarce and disseminated in a matrix of other plant species. Then, when *C. monspeliensis* cover increases, *Ps* populations tend to dilute over the resource.

To our knowledge, Corsica is the only region where *Ps* population size shows such a pattern of strong association with *C. monspeliensis* density, although this plant is widely distributed across the Mediterranean region^59^. The availability of *C. monspeliensis* in mesomediterranean habitats of Corsica might primarily explain this unusual relationship given that *Ps* is highly polyphagous. The observed preference may be driven by retained chemical signal in generations of *P. spumarius* succeeding on *C. monspeliensis* as already observed in other taxa^60^, and/or by specific communities of endosymbionts^61^. The question remains opened and more investigations are required to understand the determinisms of the association observed in Corsica (e.g., tests of host preferences in controlled conditions). In the light of this study and a previous one^28^, assessing the consequences of pulling away *C. monspeliensis* from the direct vicinity of agricultural areas as a risk mitigation strategy against *Xf* appears relevant and so seems the release of biocontrol agents^62^ near *C. monspeliensis* bushes.

*C. monspeliensis*-dense areas favoring larger populations of *Ps* and dilution over the resource may have contributed to the spread of *Xf* over Corsica^5,7^. Indeed, *C. monspeliensis* can be infected by *Xf*^63,64^ and is very abundant in early stages of mesomediterranean vegetation series in Corsica^39^. It is noteworthy that *C. monspeliensis* falls well within the description of the "hidden reservoir" suggested by epidemiological modelling^65^. *C. monspeliensis* being a pyrophyte^66^ and an early colonizer^39^, fire breaks and land clearings may have contributed to the spread of *Xf* in Corsica by increasing cover of *C. monspeliensis*. The strong association between *Ps* and *C. monspeliensis* in mesomediterranean habitats might contribute to explain why we did not observe any major outbreaks on plants sensitive to the strains of *Xf* ssp. *multiplex* present in Corsica so far^63^.

Populations of adults of *Ps* were larger in cooler and moister habitats with lower seasonal fluctuations, whatever the sampling month, and this pattern was similar whatever the type of climate data used (i.e., bioclimatic variables or seasonal raw data). This result was expected from the physiological requirements of *Ps*^2,11,12^ and from observations made in Italian grasslands, where larger populations of *Ps* are found in cooler sites^37^. This also fits with species modelling approaches, which predict low suitability for hot and dry areas in Mediterranean regions^5,6^ and is consistent with results of Avosani et al.^38^**,** who highlighted that the probability of observing *Ps* decreased when the average number of sunlight hours increased. It is noteworthy that the correlation between nymph abundance and either PC1_bioclim_ or PC1_seasonal_ was not significant, which could be the result of non-restrictive climatic conditions at sampling sites in April. The number of overwintering eggs is not well predicted by the number of gravid females, but depends primarily on weather conditions during the oviposition period^67^. Oviposition and winter survival of eggs add variability to population dynamics and may blur the climate effect found at adult stages.

Interestingly, the slope of the correlation with PC1_bioclim_ and PC1_seasonal_ was steeper in June than in October. Furthermore, local or surrounding vegetation structure had effect only on the abundance of young adults when bioclimatic data were used, with larger populations observed in plots with high scrubland vegetation and surrounded by high scrubland landscapes (tree cover > 25%). This pattern could be explained by the migration to summer hosts^17,29^ outside of the sampling plot in hotter and drier low scrubland places and a microclimatic effect benefiting *Ps* in higher scrubland vegetation^9^. Apart from this situation, local and landscape vegetation structures had no effect on *Ps* populations and *C. monspeliensis* cover was much more important to explain *Ps* abundance than broad vegetation structure.

We believe that our results can help designing surveillance plans for *Xf* that are adapted to the specificities of Corsica. Surveillance may indeed target *C. monspeliensis*-rich habitats and use *Ps* sampled there as sentinel insects to track *Xf* in the environment^5,7^. As they highlight an unexpected and unique association pattern between *Ps* and *C. monspeliensis*, our results call for more studies on the ecology of *Xf* vectors in Europe, including common ones, to better understand, anticipate and control the spread of *Xf* in a climatically changing world. So far, only a few studies have investigated spatial distribution of vectors across European semi-natural landscapes^9,37,68^. In Mediterranean regions, studies principally focused on agroecosystems and targeted only a few species of potential vectors of *Xf*. They were primarily conducted in Italian olive groves, which is explained by the dramatic economic and socio-cultural consequences of *Xf* in this area^69^. A few were also performed in almond orchards, citrus orchards or vineyards in other Mediterranean areas^16,28,70^. Yet, only studies that attempt to decipher plant-vector trophic networks or investigate the main drivers of local abundance of vectors will help to better anticipate future outbreaks and provide clues on how to manage this bacterium with sustainable practices.

## Supplementary Information

Supplementary Information 1.

Supplementary Information 2.

Supplementary Table S1.

## Data Availability

The data sets generated and analyzed during the current study are available in Table S1.
