# Supplementary material for: Environmental factors driving the abundance of Philaenus spumarius in mesomediterranean habitats of Corsica (France)
Source: Sci Rep. 2023 Feb 2;13:1901. doi: 10.1038/s41598-023-28601-4 (PMC9893205; doi:10.1038/s41598-023-28601-4)
Supplement: Supplementary file 1 — Supplementary Information 1. [file 41598_2023_28601_MOESM1_ESM.pdf]

Supplementary Information to:

Environmental factors driving the abundance of *Philaenus spumarius* in mesomediterranean habitats of Corsica (France).

Marguerite Chartois[1,2]*, Xavier Mesmin[1,2], Ileana Quiquerez[3], Sabrina Borgomano[3], Pauline Farigoule[1,4], Éric Pierre[1], Jean-Marc Thuillier[1], Jean-Claude Streito[1], François Casabianca[5], Laetitia Hugot[3], Jean-Pierre Rossi[1£], Jean-Yves Rasplus[1£] and Astrid Cruaud[1£]

[1] CBGP, INRAE, CIRAD, IRD, Institut Agro, Univ Montpellier, Montpellier, France
[2] AGAP, INRAE, CIRAD, Institut Agro, Univ Montpellier, San Giuliano, France
[3] Conservatoire Botanique National de Corse, Office de l'Environnement de la Corse, Corte, France
[4] AgroParisTech, Paris, France
[5] Centre INRAE de Corse, San Giuliano, France

£These authors are joint senior authors on this work.
*Corresponding author: marguerite.chartois@inrae.fr

Table of contents

Supplementary Figures 2

Supplementary Map 6

Supplementary Tables 6

References..7

Supplementary Figures

Fig. S1 Number of nymphs collected per foam. Black points and errorbars respectively depict the mean and 95% confidence interval (bootstrap with "stat_summary" function) of the number of foams per plot and the red horizontal line depicts the grand mean, all plots combined. A Kruskal-Wallis[1] test (number of foams ~ plot) yielded a p-value < 0.001 indicating that at least two plots differed clearly from one another. However, the Dunn test[2] classically used afterwards[3] to compare multiple means yielded no p-value below 0.09. We therefore deduced that it was not possible to distinguish clearly one plot from another in terms of nymph density per foam and so that the correlation between the number of foams and the number of nymphs was similar in all plots, i.e. $N_{nymphs} = 1.24 \times N_{foams}$.

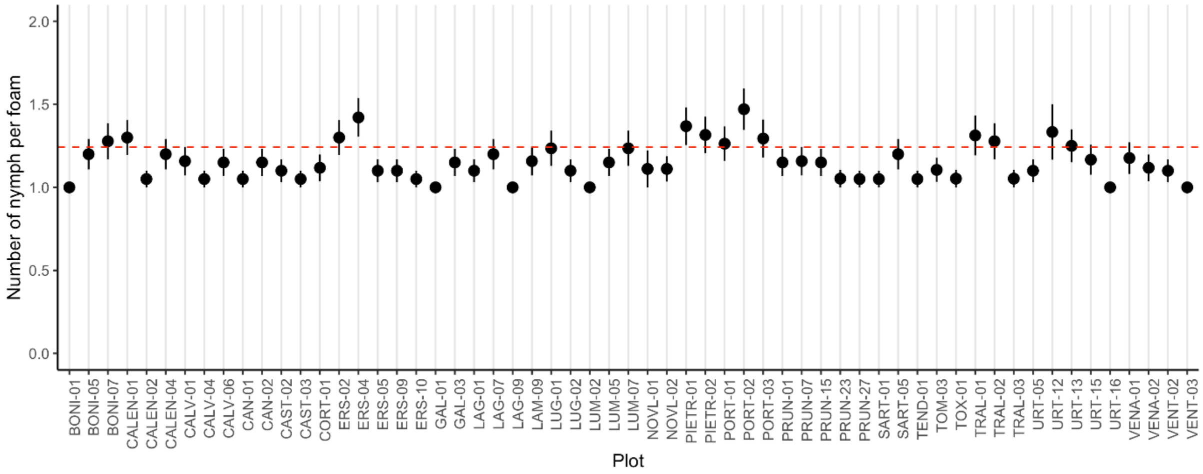

Fig. S2 Scatterplots of raw data and GLMM1 prediction for *Philaenus spumarius* abundance and year by sampling month. A: April. **B:** June. **C**: October.

Error bars on scatterplots: 95% confidence intervals. Letters: pairwise comparison of estimated marginal means; by panel, distributions sharing a letter do not differ significantly. Points: raw data (provided in Table S1).

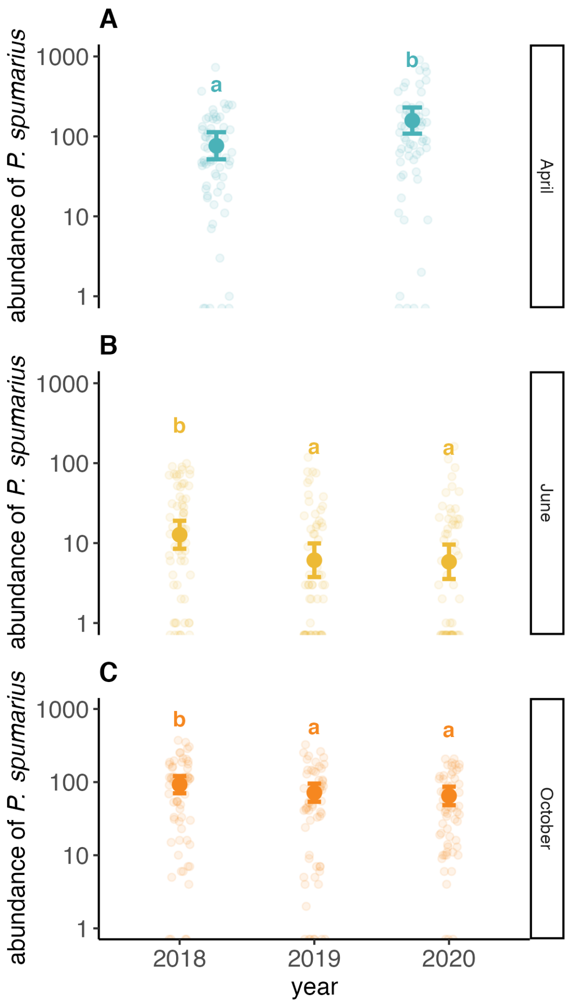

Fig. S3 Principal component analyses of the 10 seasonal climate descriptors.

Left: Graphic of variables. **Right**: Graphic of sampling plots. **AB:** April. **CD:** June. **EF:** October. Raw data are provided in Table S1.

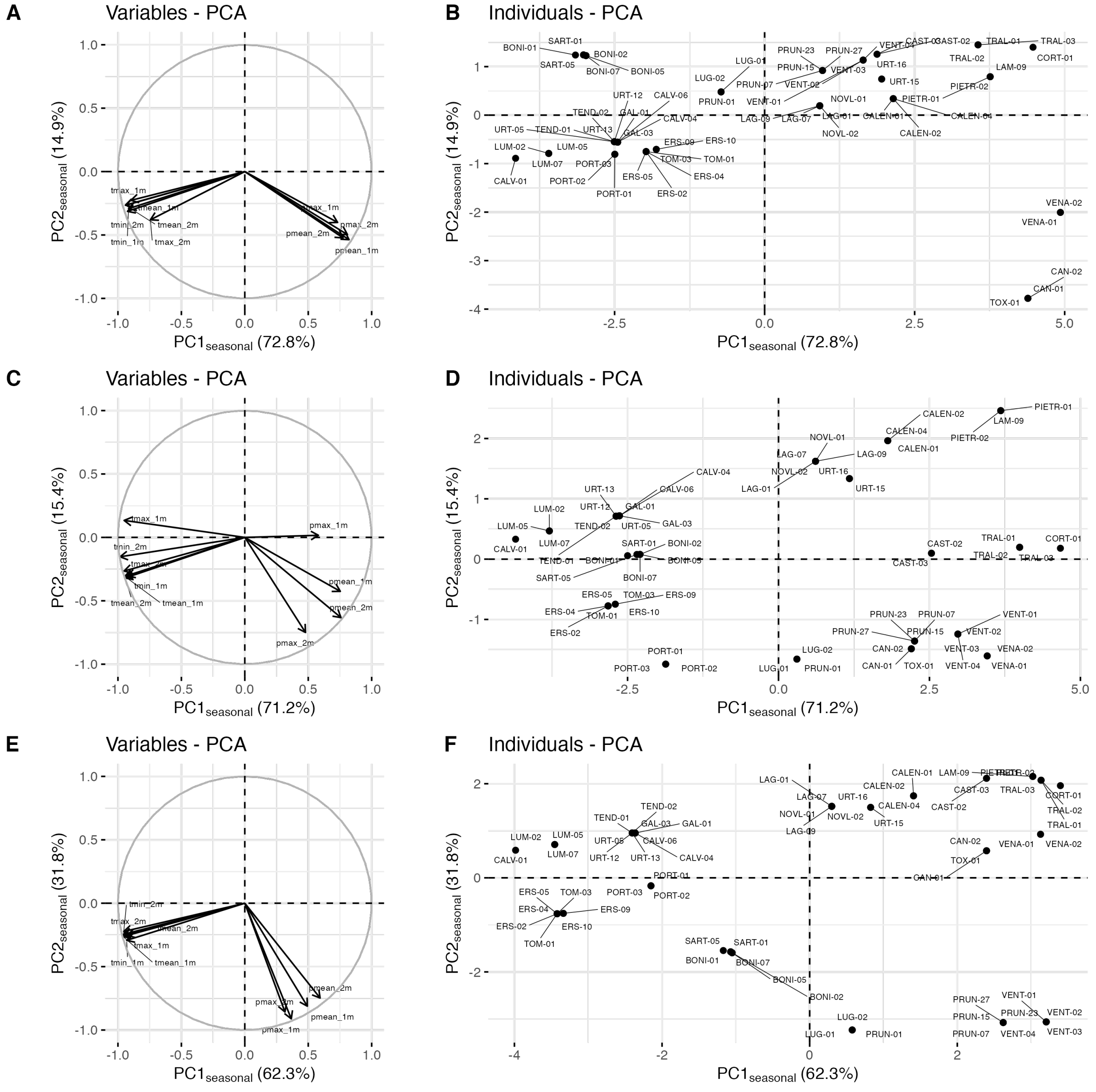

Fig. S4 Scatterplots of raw data, regression lines and model prediction for pairwise associations between *P. spumarius* (*Ps*) abundance and fixed effects (GLMM1bis). A: *P. spumarius* abundance and percent cover of *Cistus monspeliensis*. **B:** *P. spumarius* abundance and plot scores on $PC1_{seasonal}$. Whatever the sampling season, $PC1_{seasonal}$ opposed plots with high precipitations and low temperatures (right side of the axis) to plots with high temperatures and low precipitations (left side of the axis). **C:** *P. spumarius* abundance and plot scores on $PC2_{seasonal}$). Whatever the season, $PC2_{seasonal}$ opposed plots with low precipitations and low temperatures (top side of the axis) to plots with high temperatures and high precipitations (bottom side of the axis). **D:** *P. spumarius* abundance and year by sampling month. Colors: sampling month. Error bars on scatterplots: 95% confidence intervals. Letters: pairwise comparison of estimated marginal means; by panel, distributions sharing a letter do not differ significantly. Points: raw data (provided in Table S1); lines: regression curves from models. All correlations have p-values < 0.05. Full details on significance tests are given in Table S2.

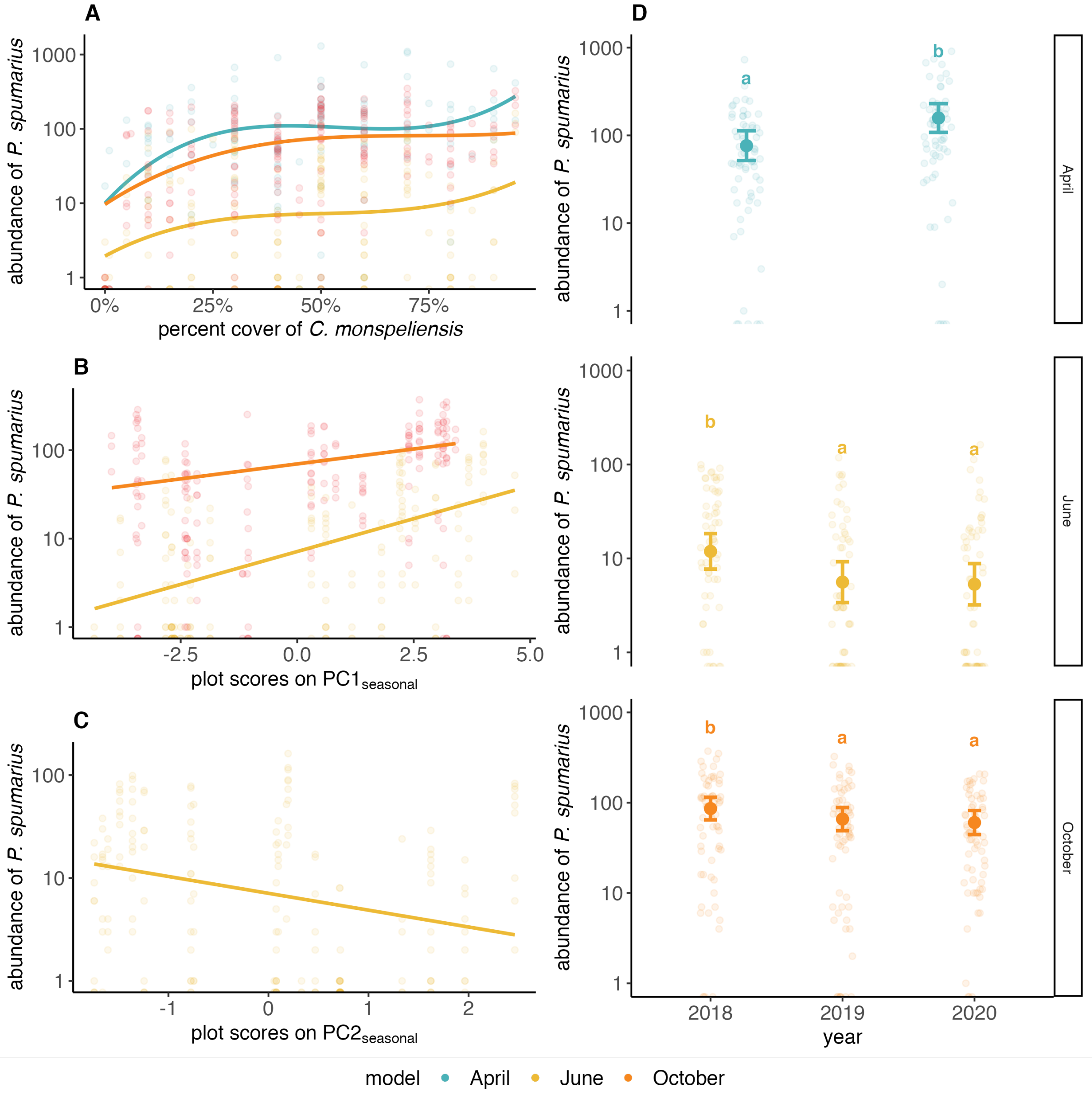

Supplementary Map

Distribution of sampling plots and their local and landscape vegetation structure. Downloadable in ".html" format in online Supplementary Materials.

Supplementary Tables

Table S1 Raw data collected during this study and analyzed in this article. Downloadable in ".xlsx" format in online Supplementary Materials.

Table S2 Values and significance of Type-II Wald chi square tests performed on GLMM1, GLMM1bis, and GLMM2 models. df = degree of freedom. Not significant fixed effects are figured with a dash.

	**APRIL**			**JUNE**			**OCTOBER**		
**Model**	GLMM1-April			GLMM1-June			GLMM1-October		
**Marginal R^2**	0.41			0.64			0.65		
**Fixed effects**	χ^2	**df**	**P-value**	χ^2	**df**	**P-value**	χ^2	**df**	**P-value**
%cover of *C. monspeliensis*	25.242	3	1.375e-05	15.682	3	1.318e-03	40.341	3	9.021e-09
vegetation structure	-	-	-	4.149	1	4.166e-02	-	-	-
plot scores on $PC1_{bioclim}$	-	-	-	38.216	1	6.333e-10	15.569	1	7.954e-05
plot scores on $PC2_{bioclim}$	-	-	-	12.623	1	3.811e-04	9.840	1	1.707e-03
plot scores on $PC1_{landscape}$	-	-	-	4.862	1	2.745e-02	-	-	-
plot scores on $PC2_{landscape}$	-	-	-	-	-	-	-	-	-
year	23.857	1	1.038e-06	29.298	2	4.345e-07	13.270	2	1.313e-03
	**APRIL**			**JUNE**			**OCTOBER**		
**Model**	GLMM1bis-April			GLMM1bis-June			GLMM1bis-October		
**Marginal R^2**	0.41			0.53			0.57		
**Fixed effects**	χ^2	**df**	**P-value**	χ^2	**df**	**P-value**	χ^2	**df**	**P-value**
%cover of *C. monspeliensis*	25.242	3	1.375e-05	10.377	3	1.562e-02	34.907	3	1.274e-07
plot scores on $PC1_{seasonal}$	-	-	-	31.979	1	1.559e-08	15.623	1	7.732e-05
plot scores on $PC2_{seasonal}$	-	-	-	9.711	1	1.831e-03	-	-	-
plot scores on $PC1_{landscape}$	-	-	-	-	-	-	-	-	-
plot scores on $PC2_{landscape}$	-	-	-	-	-	-	-	-	-
vegetation structure	-	-	-	-	-	-	-	-	-
year	23.857	1	1.038e-06	33.622	2	5.000e-08	14.072	2	8.797e-04
	**APRIL**			**JUNE**			**OCTOBER**		
**Model**	GLMM2-April			GLMM2-June			GLMM2-October		
**Marginal R^2**	0.29			0.72			0.52		
**Fixed effects**	χ^2	**df**	**P-value**	χ^2	**df**	**P-value**	χ^2	**df**	**P-value**
%cover of *C. monspeliensis*	15.757	3	1.272e-03	22.183	3	5.975e-05	21.363	3	8.850e-05
plot scores on $PC1_{bioclim}$	-	-	-	42.774	1	6.146e-11	15.645	1	7.643e-05
plot scores on $PC2_{bioclim}$	-	-	-	12.992	1	3.128e-04	9.483	1	2.074e-03
plot scores on $PC1_{landscape}$	-	-	-	7.204	1	7.275e-03	-	-	-
plot scores on $PC2_{landscape}$	-	-	-	-	-	-	-	-	-
vegetation structure	-	-	-	5.704	1	1.693e-02	-	-	-
year	23.262	1	1.414e-06	27.937	2	8.583e-07	13.808	2	1.004e-03

Table S3 Climate profile of the most contrasted plots according to PCA performed on the 19 bioclimatic variables.

		**Plot representative of low temperature and high precipitations**	**Plot representative of high temperature and low precipitations**
**Variables with high contribution to $PC1_{bioclim}$**	Annual mean temperature	12.5°C	17.3°C
	Precipitations of wettest month	208.9 mm	101.6 mm
		**Plot representative of high contrasted seasons**	**Plot representative of low contrasted seasons**
**Variables with high contribution to $PC2_{bioclim}$**	Daily temperature range	8.6°C	7.3°C
	Annual temperature range	24.6°C	22.5°C

References

1. Hollander, M., Wolfe, D. A. & Chicken, E. Nonparametric Statistical Methods. (John Wiley & Sons, Inc., 2014).
2. Dunn, O. J. Multiple Comparisons Using Rank Sums. Technometrics 6, 241–252 (1964).
3. Hervé, M. Aide-mémoire de statistique appliquée à la biologie. (2016).
