# Supplementary material for: Environmental factors driving the abundance of Philaenus spumarius in mesomediterranean habitats of Corsica (France)
Source: Sci Rep. 2023 Feb 2;13:1901. doi: 10.1038/s41598-023-28601-4 (PMC9893205; doi:10.1038/s41598-023-28601-4)
Supplement: Supplementary file 2 — Supplementary Information 2. [file 41598_2023_28601_MOESM2_ESM.html]

Supplementary map to Environmental factors driving the abundance of Philaenus spumarius in mesomediterranean habitats of Corsica (France)


# Supplementary map to Environmental factors driving the abundance of *Philaenus spumarius* in mesomediterranean habitats of Corsica (France)

Marguerite Chartois, Xavier Mesmin, Ileana Quiquerez, Sabrina Borgomano, Pauline Farigoule, Éric Pierre, Jean-Marc Thuillier, Jean-Claude Streito, François Casabianca, Laetitia Hugot, Jean-Pierre Rossi, Jean-Yves Rasplus and Astrid Cruaud

**Distribution of sampling plots and their local and landscape vegetation structure.** Perimeter of plots is delimitated by a black border. The dominant local vegetation structure within plots borders is symbolized by the color code of markers. Landscape vegetation structure is described within 374 meters radius buffers zones around plots.

Vegetation structure was retrieved from the OCS GE database (© IGN – 2022, https://geoservices.ign.fr/ocsge) provided by the French National Institute of Geographic and Forestry Information.
